# The Application of Heptamethine Cyanine Dye DZ-1 and Indocyanine Green for Imaging and Targeting in Xenograft Models of Hepatocellular Carcinoma

**DOI:** 10.3390/ijms18061332

**Published:** 2017-06-21

**Authors:** Caiqin Zhang, Yong Zhao, He Zhang, Xue Chen, Ningning Zhao, Dengxu Tan, Hai Zhang, Changhong Shi

**Affiliations:** Laboratory Animal Center, the Fourth Military Medical University, Xi’an 710032, China; zhangcaiqin-beibei@163.com (C.Z.); zhaoyong8@aliyun.com (Y.Z.); alwayszhh@163.com (H.Z.); chenxuefamily6@163.com (X.C.); 18729517815@163.com (N.Z.); dgjdg@foxmail.com (D.T.); hzhang@fmmu.edu.cn (H.Z.)

**Keywords:** near-infrared fluorescence, heptamethine cyanine dyes, hepatocellular carcinoma, xenograft model, optical imaging, intraoperative navigation

## Abstract

Near infrared fluorescence (NIRF) imaging has strong potential for widespread use in noninvasive tumor imaging. Indocyanine green (ICG) is the only Food and Drug Administration (FDA) -approved NIRF dye for clinical diagnosis; however, it is unstable and poorly targets tumors. DZ-1 is a novel heptamethine cyanine NIRF dye, suitable for imaging and tumor targeting. Here, we compared the fluorescence intensity and metabolism of DZ-1 and ICG. Additionally, we assayed their specificities and abilities to target tumor cells, using cultured hepatocellular carcinoma (HCC) cell lines, a nude mouse subcutaneous xenograft model of liver cancer, and a rabbit orthotopic transplantation model. We found that DZ-1 accumulates in tumor tissue and specifically recognizes HCC in subcutaneous and orthotopic models. The NIRF intensity of DZ-1 was one order of magnitude stronger than that of ICG, and DZ-1 showed excellent intraoperative tumor targeting in the rabbit model. Importantly, ICG accumulated at tumor sites, as well as in the liver and kidney. Furthermore, DZ-1 analog-gemcitabine conjugate (NIRG) exhibited similar tumor-specific targeting and imaging properties, including inhibition of tumor growth, in HCC patient-derived xenograft (PDX) mice. DZ-1 and NIRG demonstrated superior tumor-targeting specificity, compared to ICG. We show that DZ-1 is an effective molecular probe for specific imaging, targeting, and therapy in HCC.

## 1. Introduction

Near-infrared fluorescence (NIRF) imaging is a promising tool for noninvasive tumor imaging [1,2]. Since tissues exhibit almost no autofluorescence in the NIR spectrum, the signal-to-background ratio can be maximized using NIRF contrast agents. These NIRF agents can be readily detected within deep tissues by imaging modalities and are effective for detection of tumor locations [3]. NIRF agents are characterized by high extinction coefficients and relatively large Stokes’ shifts [4]. They have shown great potential for both experimental and clinical imaging [5,6], including real-time visualization of certain anatomical structures during surgery [7,8]. Many NIRF agents, such as Cy5.5 and IR800-CW, have been successfully used for diagnosis in tumor models after labeling with an experimental peptide or a specific antigen [9,10]. A representative NIRF agent is indocyanine green (ICG), which is the only FDA-approved NIRF dye for clinical diagnosis. However, the stability of ICG is poor, and its rapid clearance rate in plasma limits the application of ICG in tumor fluorescence imaging [11].

Our research group has synthesized and screened a series of heptamethine cyanine NIRF dyes, including IR-783 and MHI-148 [12,13]. These compounds displayed tumor targeting capabilities, in both tumor cells and various nude mouse xenograft models [14,15]. These dyes were also conjugated with a variety of antitumor drugs to target cancer cells; this allowed real-time monitoring of therapeutic effects and reduced drug dosages, avoiding the potential for side effects [16,17]. However, the water solubility of these dyes is poor—all must be dissolved in dimethyl sulfoxide and then diluted with phosphate-buffered saline for intraperitoneal injection during in vivo imaging applications [18]. To improve the water solubility of the NIRF compound MHI-148, we replaced one of its side chains, (CH_2_)_5_CO^2−^, with (CH_2_)_4_SO^3−^. This modified compound, DZ-1, is water-soluble and can be injected intravenously for in vivo imaging applications.

Hepatocellular carcinoma (HCC) is a high incidence tumor that requires imaging-based early detection for effective clinical management and therapeutic outcomes. In this study, we established a nude mouse subcutaneous xenograft model of HCC and a rabbit orthotopic transplantation model of HCC. Through these models, we compared the fluorescence intensity and metabolism of DZ-1 and ICG. Additionally, we assayed specificity, targeting, and preferential uptake by the tumor cell, in order to test the feasibility of replacing ICG with DZ-1. Moreover, the novel compound, DZ-1 conjugated with chemotherapeutic drugs gemcitabine (NIRG) [13], was evaluated in the HCC xenograft mouse model for imaging, targeting, and therapeutic applications.

## 2. Results

### 2.1. Uptake of ICG and DZ-1 by HCC

**Figure 1:**
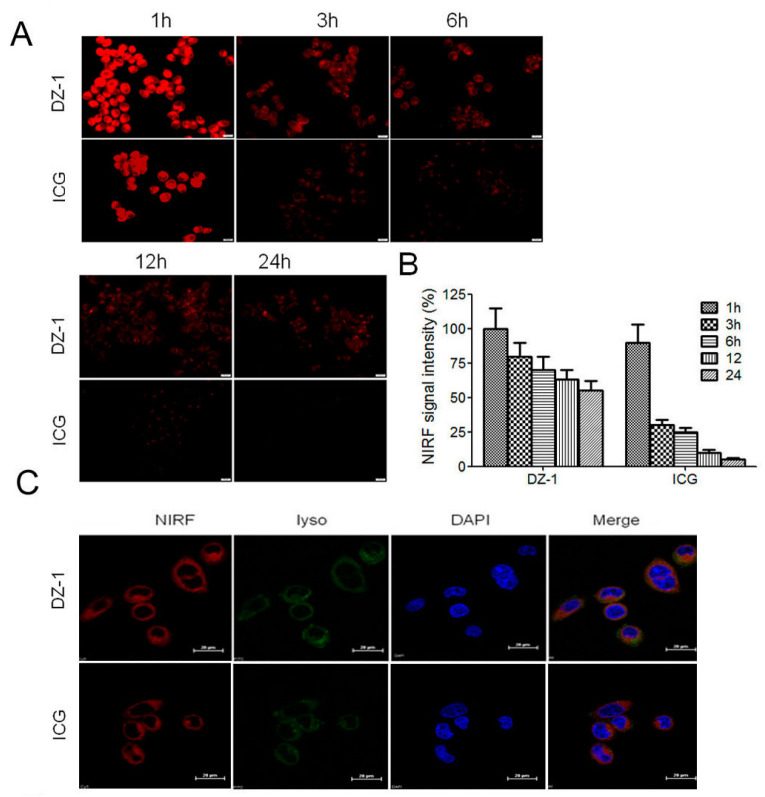

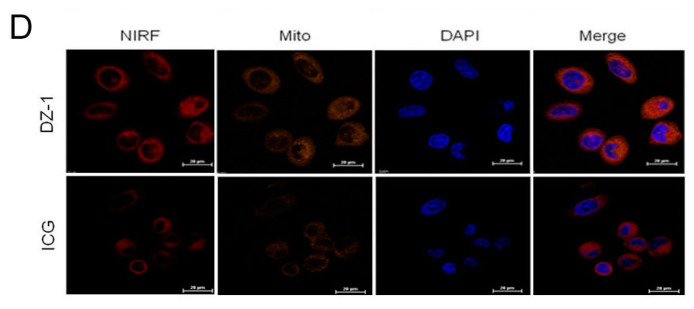
Uptake of DZ-1 and indocyanine green (ICG) in hepatocellular carcinoma (HCC). (**A**) Confocal microscope analyses of DZ-1 dye uptake by HCC. Original magnification: 400×; scale bars: 50 μm; (**B**) Ratio of near infrared fluorescence (NIRF, **red**) dye uptake intensity in HCC at successive time points. Data are presented as mean ± standard deviation (SD) (*n* = 5); (**C**,**D**) Co-localization of DZ-1 or ICG (**red**) and LysoTracker (**green**, **top** panel) or MitoTracker (**yellow**, **bottom** panel) in Hep3B cells, as determined by confocal microscopy. Cell nuclei were stained with 4′,6-diamidino-2-phenylindole (DAPI, **blue**). Original magnification: 1200×; Scale bars, 20 μm.

After ICG or DZ-1 (20 μM) was co-incubated with cultured human HCC cell line Hep3B2.1-7 (Hep3B) for 30 min, and washed with PBS. NIRF dye uptake in HCC was analyzed at successive time points by a NIRF microscope. We found that the NIRF intensity of tumor cells was the strongest at 1 h; the intensity decreased after 3 h, and the dye metabolism began to accelerate. At 12 and 24 h, cells treated with DZ-1 dye still emitted fluorescent signals. However, the fluorescence intensity of cells treated with ICG decreased significantly after 3 h and was considerably weaker than DZ-1. At 24 h, almost no fluorescence signal could be detected from the ICG dye, while cells treated with DZ-1 dye still emitted 50% of fluorescent signals (Figure 1A,B).

To determine the preferential organelle(s) for NIRF dyes at the cellular level, Hep3B cells were seeded on petri dishes for 24 h, then exposed to MitoTracker (200 nM) or LysoTracker (75 nM) at 37 °C for 30 min. DZ-1 or ICG (20 μM) was added, and cells were incubated for 30 min at 37 °C. The uptake of dyes was determined by microscopic examination as described above. As shown in Figure 1C, DZ-1 co-localized with MitoTracker (yellow) or LysoTracker (green) in Hep3B cells, indicating that DZ-1/ ICG is primarily retained within mitochondria and lysosomes.

### 2.2. Organ Distribution of ICG and DZ-1 in Nude Mouse Model of HCC

**Figure 2:**
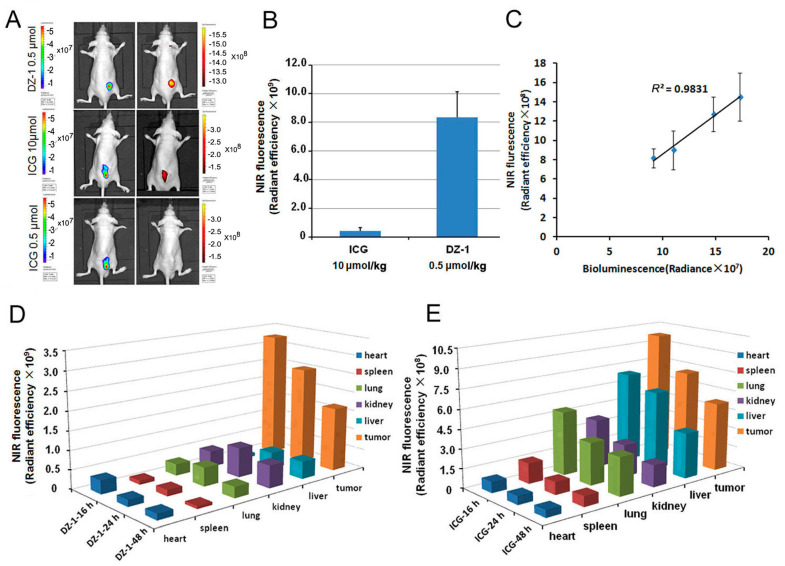
The distribution of DZ-1 and ICG in the organs of subcutaneous tumor model. (**A**) Dual ex vivo BLI/NIRF imaging of mice with Hep3B-Luc subcutaneous xenografts treated with varying doses of ICG or DZ-1; (**B**) Quantification of NIRF intensity within the tumor area (per cm^2^) of subcutaneous tumor xenografts; (**C**) NIRF/BLI signal intensity correlation in mice (*n* = 5) with subcutaneous tumor xenografts (right); (**D**,**E**) Distribution intensity per cm^2^ of DZ-1 and ICG in the organs of subcutaneous tumor models at successive times.

The luc-tagged Hep3B cells were inoculated subcutaneously into nude mice, and tumors formed after two weeks. ICG was injected intravenously into mice bearing tumors, at a dose of 10 μmol/kg. After 24 h, the tumor location was determined by NIRF optical imaging, and the fluorescence intensity within the tumor area (per cm^2^) was about 4.14 × 10^8^. We only detected weak fluorescence after the injection of ICG at a dose of 0.5 μmol/kg, while the injection of DZ-1 at a dose of 0.5 μmol/kg yielded a fluorescence intensity within the tumor area (per cm^2^) of approximately 8.35 × 10^9^ (Figure 2A,B).

We further intravenously injected DZ-1 (0.5 μmol/kg) into nude mice bearing different numbers (1 × 10^6^, 3 × 10^6^, 6 × 10^6^ and 9 × 10^6^) of subcutaneously inoculated luc-tagged Hep3B tumor cells. These mice were subjected to whole-body optical imaging by co-registered bioluminescence (BLI) and NIRF signals within the tumors. Both NIRF signals and Bioluminescence (BLI) increased with tumor growth, and showed a significant positive association between the two imaging modalities (*R*^2^ = 0.9831; Figure 2C).

The mice bearing subcutaneous HCC tumors were injected with ICG (10 μmol/kg) or DZ-1 (0.5 μmol/kg), and sacrificed at various time points to detect NIRF intensity per cm^2^ of major organs (Figure 2D,E). DZ-1 demonstrated high specificity in tumor tissue at 16, 24, and 48 h. However, ICG signals accumulated not only at the tumor position, but also in liver and lung tissue. Moreover, the specificity of ICG fluorescence intensity in tumor tissue was poor.

### 2.3. Metabolic Characteristics of ICG/DZ-1 in Nude Mice with Subcutaneous HCC Xenograft

**Figure 3:**
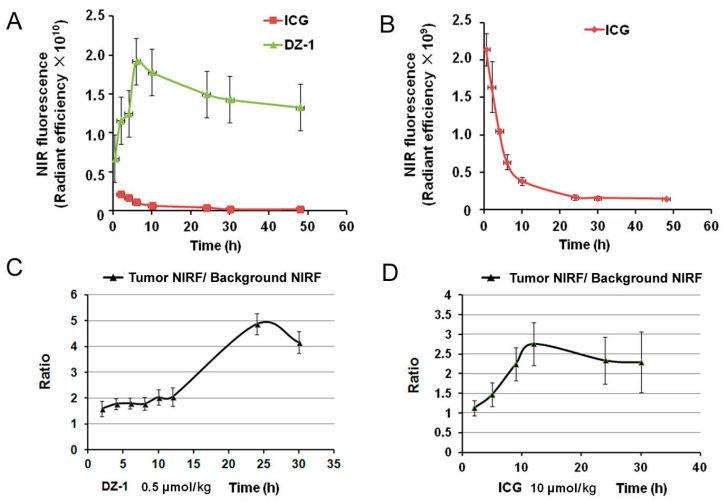
Metabolism of DZ-1 and ICG in the organs of subcutaneous tumor model. (**A**,**B**) NIRF intensities per unit area (cm^2^) in subcutaneous tumor xenograft mice subjected to DZ-1 or ICG were measured at 0.5, 2, 3, 6, 10, 24, and 48 h; (**C**,**D**). Ratio of tumor NIRF intensity to background NIRF intensity at various time points after injection of DZ-1 or ICG in subcutaneous tumor xenograft mice.

We also determined NIRF intensity value per cm^2^ of the tumor site (Tumor, T), after injection of ICG or DZ-1 as described above, to obtain the NIRF intensity value per cm^2^ of normal tissue (Background, B). After DZ-1 injection into nude mice, the fluorescence intensity increased, became stable by 6 h, and reached a plateau at 24 h (Figure 3A). The ratio of tumor intensity to background intensity (T/B) increased gradually, and reached its peak at 24 h (Figure 3C), where the average value was approximately 5.0. When ICG was injected into nude mice bearing HCC tumors, the fluorescence intensity at the tumor site reached its peak at 0.5 h, and then decreased rapidly. At 12 h, the tumor intensity maintained some stability, but the background value declined rapidly, thus inducing the largest T/B value, an average of 2.5. This suggests that the metabolism of ICG is faster than DZ-1 in vivo (Figure 3).

### 2.4. Imaging of ICG/DZ-1 in Nude Mice with Orthotopic HCC Xenograft

**Figure 4:**
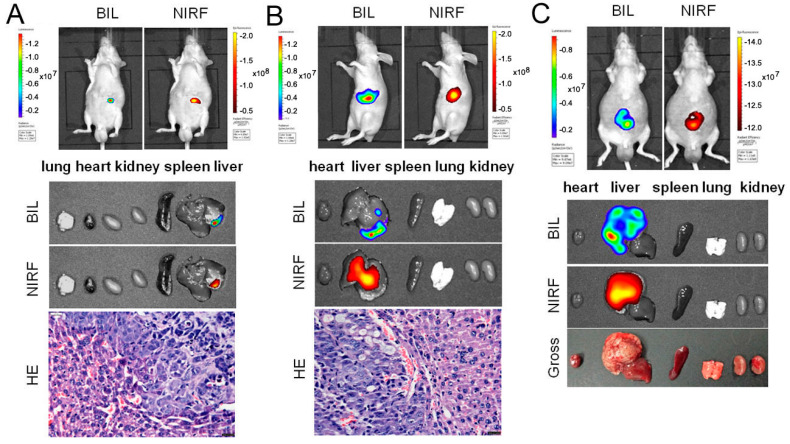
Comparison of ICG and DZ-1 imaging in orthotopic liver tumors. (**A**) Representative NIRF optical images of tumor sites by injection of DZ-1 (0.5 μmol/kg) in vivo. Scale bar: 20 μm. (**B**) Representative NIRF optical images of tumor sites by injection of ICG (10 μmol/kg) in vivo. However, there were more differences between the bioluminescence signal range and fluorescence in the tumor site in vitro. Scale bar: 20 μm. (**C**) A large area of carcinogenesis of liver identified by ICG (10 μmol/kg). The ex vivo signal range of bioluminescence in the organ is consistent with that of fluorescence.

Both ICG (10 μmol/kg) and DZ-1 (0.5 μmol/kg) were intravenously injected into orthotopic liver transplant model with Hep3B-Luc cells. The NIRF signal of the tumor site could be detected in the liver of nude mice by a small animal optical imaging system (Figure 4A,B). After anatomy by ex vivo NIRF imaging, we found that ICG had mainly accumulated in the tumor within the hepatic portal area, and that the tumor within the hepatic margin was difficult to detect. Most of the portions of the liver with carcinogenesis could be identified. In contrast, DZ-1 was effective for the identification of smaller tumor regions in the liver (Figure 4).

### 2.5. Intraoperative Navigation of Rabbit Liver Cancer Model by ICG/DZ-1

**Figure 5:**
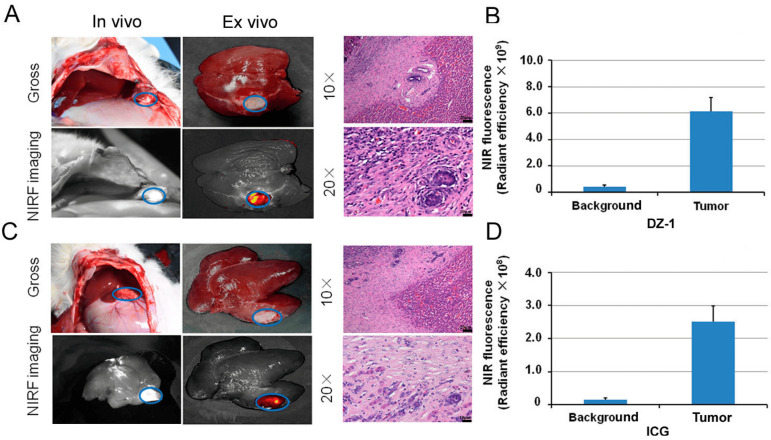
The application of DZ-1 and ICG in surgical exploration. (**A**) DZ-1 imaging using NIRF optical fiber intraoperative guidance in rabbits with VX2 liver cancer (**left**). Ex vivo NIRF imaging of rabbit liver cancer using small animal optical imaging system (**right**). Hematoxylin-eosin (H&E) staining of the fluorescent tissue sections; (**B**) NIRF intensity/tumor area (per cm^2^) of DZ-1 uptake in liver tumor of rabbit; (**C**) ICG imaging using NIRF optical fiber intraoperative guidance in rabbits with VX2 liver cancer (**left**). Ex vivo NIRF imaging of rabbit liver cancer using small animal optical imaging system (**right**). H&E staining of the fluorescent tissue sections. 10× and 20×, Scale bars, 20 μm; (**D**) NIRF intensity/tumor area (per cm^2^) of ICG uptake in liver tumor of the rabbits. Blue circle indicate the tumor location.

Rabbits bearing a VX2 orthotopic liver transplantation tumor were injected with ICG and DZ-1, and then subjected to surgical exploration. The NIRF signal could be detected in the rabbit liver lobe under NIRF optical fiber exploration, consistent with the gross observation of the transplantation tumor (Figure 5A,C, left). The NIRF signal of the tumor site could also be found using the small animal optical imaging (Figure 5A,C, right), and the fluorescence intensity/tumor area (F/T) after DZ-1 injection was much higher than that of the ICG injection group (Figure 5B). Although the injection dose of DZ-1 was only 1/20 of that of ICG, the Fluorescence intensity/tumor area (F/T) value reached 6.11 × 10^9^, while in the ICG group, it was only 2.51 × 10^8^ (Figure 5B,D). Hematoxylin-eosin (H&E) staining of the fluorescent sections showed obvious morphological characteristics of liver cancer, and the pathological results were consistent with the results of surgical exploration and in vivo imaging (Figure 5).

### 2.6. DZ-1-Mediated Targeted Delivery of Antitumor Drugs

**Figure 6:**
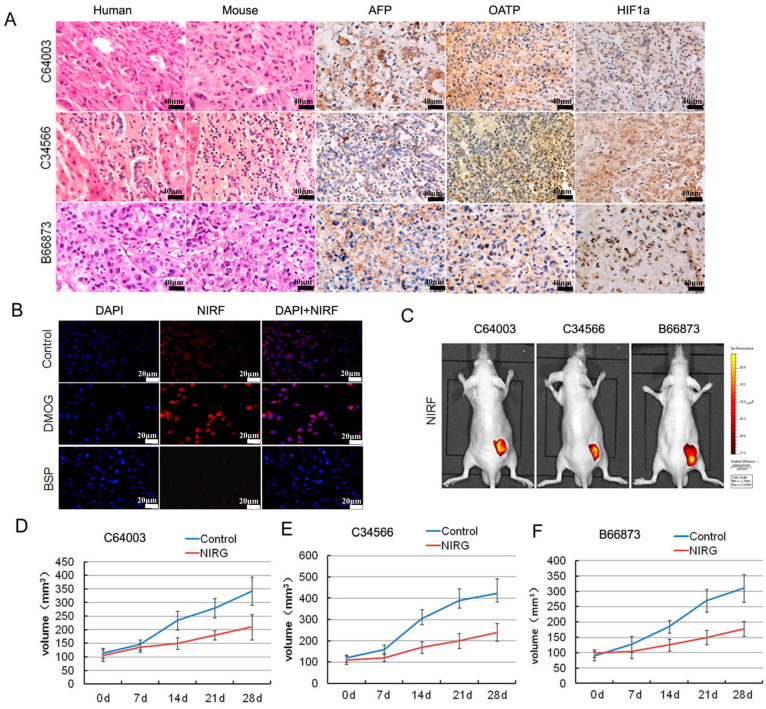
Imaging and targeting of DZ-1 drug conjugate in a liver cancer patient-derived xenograft (PDX) model. (**A**) H&E, NIRF, and immunohistochemistry (IHC) analyses of liver cancer tissues derived from both PDX mouse models and original patient samples. Original magnification: 400×; scale bars represent 20 μm; (**B**) DZ-1 dye uptake by Hep3B cells with a prior exposure to either HIF1α stabilizers (DMOG), or OATP inhibitor (BSP). Scale bar, 50 μm; (**C**) NIRF optical imaging of PDX models established by implanting 3 different human liver cancer specimens to subcutaneous of nude mice. Strong fluorescent signal was detected at subcutaneous tumor site; (**D**–**F**) Inhibition of NIRG on the tumor growth from liver cancer PDX mouse, including C64003, C34566, and B66873. d represents the treatment time.

Three PDX models were successfully established by transplanting fresh human liver cancer specimens into nude mice subcutaneously. The histology of patient-derived xenograft (PDX)-derived tumor tissues was identical to the original patient’s tumor samples by H&E staining, which was clearly defined by the strong expression of α-fetal protein (AFP), which were well-expressed markers in HCC (Figure 6A). We also detected hypoxia-inducible factor 1-α (HIF1α) and organic anion-transporting polypeptides (OATP) expression (Figure 6A), which are considered to increase dye uptake in cancer cells in different tissue samples [13,19]. To determine whether the specific uptake and accumulation of NIR dye in liver cancer cells is mediated by HIF1α/OATP, Hep3B cells were pre-incubated with HIF1α stabilizers dimethyloxaloylglycine (DMOG) (1 mM, Millipore, Billerica, MA, USA) for 24 h, or with OATP inhibitor bromosulfophthalein (BSP) (250 μM, Sigma-Aldrich, St. Louis, MO, USA) for 1 h prior to DZ-1 staining and NIR fluorescence detection. After fixing with cold formalin, the cells were counterstained with 4′,6-diamidino-2-phenylindole (DAPI). As shown in Figure 6B, DMOG significantly enhanced the dye uptake in cancer cells. Conversely, BSP attenuated the uptake and retention of NIR dye by cancer cells.

The tumor tissue from different PDX models was cut into 3-mm^3^ pieces and implanted to nude mice subcutaneously (*n* = 5 for each group). Twenty days later, mice were injected intraperitoneally with either saline or NIRG (10 mg/kg) twice a week for four weeks [10]. The tumor size was measured at seven, 14, 21, and 28 days after administration of NIRG [20]. The results of whole-body NIRF imaging are shown in Figure 6C. The conjugate compound was taken up specifically by liver tumor in the mice, retaining strong NIRF signals. Furthermore, NIRG could inhibit the tumor growth in PDX mouse models significantly (Figure 6D,F). This showed the dual effects of fluorescence imaging and tumor targeted therapy.

## 3. Discussion

NIRF dye ICG can be quickly combined with plasma protein, distributed in systemic blood vessels, and efficiently and selectively taken up by liver cells with rapid blood circulation, which aids in diagnosis of liver fibrosis and cirrhosis and gastrointestinal vascular defects [21,22,23,24]. It is mainly used in the intraoperative navigation of tumor, and has good water solubility with high safety, but lacks tumor targeting [25]. The conventional NIR dye needs to be labeled with peptides or a specific antigen marker, which facilitates targeting, although only a part of the tumor can be identified, and the chemical combination often affects the stability of the target molecule [26,27]. Thus, it is urgent to explore novel compounds with dual effects for fluorescent imaging and tumor targeting. Although ICG can be used for intraoperative navigation and laparoscopic surgery for liver cancer, it has poor specificity for binding to tumor cells and can induce strong fluorescence in normal tissues. Moreover, the liver is the main organ for ICG metabolism, where it shows non-specific aggregation, which limits the application of ICG in visualization during tumor surgery [28]. Further, ICG shows poor stability, and thus, it decomposes easily in polar solution. However, in this study, heptamethine cyanine compounds displayed the characteristics of NIRF and tumor targeting, thus providing a possible approach for clinical imaging of cancer.

Although many strategies have been adopted to improve the stability and specificity of NIR dyes, the main problem is associated with the non-universal nature of the target-receptor. For example, biological receptors like EGFR, HER2, and uPAR have been reported to be abundant in some tumors, while they are less common in others [29]. An ideal imaging fluorophore probe for clinical application should possess the properties of selective accumulation, intense emission, high photo-stability, and high quantum yields [30]. To meet these challenges, researchers are now focusing on the development of new probes with improved photophysical properties, ability for localization, and stability [10].

It has been reported that tumor-specific accumulation of heptamethine cyanine dye is closely associated with the activation of HIF1α and organic anion-transporting polypeptide (OATPs) [17,18,19,31]. Hypoxia and OATP expression are common phenomena in most types of cancers [32,33]. Previous studies have demonstrated that OATP dominantly controls the transport of NIRF dyes, and hypoxia further increases dye uptake by cancer cells [19]. This clearly demonstrates the advantage of heptamethine cyanine dyes over other NIRF agents for cancer imaging. In this study, the results showed that HIF1α stabilizers increased the uptake of DZ-1 by liver cancer cells, while OATP inhibitors reduced the NIR dye uptake. Importantly, we detected intense widespread nuclear HIF1α and cytoplasmic OATP expression in tumor samples (Figure 6A), which indicates that these two molecules are involved in the preferential uptake of NIRF dyes. These clinical observations reinforce the cancer-specific targeting ability of NIRF dye as well as the roles of HIF1α/OATPs in mediating dye uptake in liver cancer. In addition to the well-elucidated hypoxia/HIF1α- and OATPs-mediated mechanisms, other alternative mechanisms might involve in the accumulation of NIRF dyes in liver cancer cells, such as IR-780 and Pz 247 [34,35]. These heptamethine cyanine dyes can achieve organelle-preferential accumulation in cancer cells by the enhanced permeability and retention (EPR) effect, which facilitates the entrance of dyes to the intracellular compartment with higher retention in the mitochondria and lysosomes through ATP-dependent endocytosis [3].

While the preferential uptake of ICG by the tumor cells is regulated by the cell membrane-binding ability of ICG, OATP1B3 and multi-drug resistance p-glycoprotein-3, as the main ICG-related transporters, play a pivotal role in the uptake of ICG in HCC; the former is an influx transporter, and the latter is an efflux transporter. Uptake of ICG by differentiated HCC cells with concomitant biliary excretion disorder was shown to lead to the accumulation of ICG in cancer tissues [36]. In addition, it was reported that portal uptake of ICG in HCC cells is mediated by the Na+/taurocholate transport protein [37]. Thus, ICG can be used for liver cancer imaging with certain site specificity. However, the specificity is better if the tumor is located at the portal site; poor specificity is observed otherwise. This is the main reason that the fluorescence imaging range and tumor location do not completely coincide in Figure 2. Regarding the tumor-to-background ratio of NIRF signals at 24 h for tumor detection in mice, DZ-1 showed a higher ratio than ICG; this provides a rationale for elucidating the advantages of the dyes used in our study over other NIRF agents for cancer imaging (Figure 3C,D).

The uptake of DZ-1 by HCC was not affected by the tumor formation site, and good specificity was observed. The signal range of bioluminescence and fluorescence imaging showed excellent coincidence. When a mouse HCC model is used for whole-body NIRF imaging, the best time of signal acquisition should be at the high fluorescence intensity of the tumor site, when the background value is low enough to form a high T/B value. According to the results obtained, the best detection time in NIRF imaging of DZ-1 in mice bearing HCC was approximately 20 h, which is consistent with the previous report [12], and that of ICG should be approximately 10 h. Comparing the distribution of DZ-1 and ICG in HCC subcutaneous transplantation nude mice, we found that DZ-1 was mainly concentrated in the tumor site, while other organs exhibited a reduced NIRF signal; moreover, it showed good tumor specificity from 12 to 48 h after the injection. However, ICG signal not only accumulated at tumor sites, but also in the liver and kidney, and presented similar fluorescence intensity with the tumor site. This nonspecific binding can persist for 48 h and form high background, leading to difficultly in distinguishing whether the tumor is in the liver or in subcutaneous tissue.

DZ-1 (0.5 μmol/kg) injection of nude mice with orthotopic HCC transplantation showed a good correlation between the signal of NIRF and BLI at tumor site by optical imaging. The signal range was similar to the tumor. While the signal of both NIRF and BLI showed the tumor location after injection of ICG (10 μmol/kg) in vivo, in ex vivo imaging, the range of the two imaging signals showed some deviation; however, when the tumor was large enough, the difference became smaller. These results suggested that the accuracy of ICG detection of liver cancer in the orthotopic model was closely related to the size and location of the tumor. In contrast, DZ-1 can identify liver cancer in orthotopic model specifically, even with smaller tumors.

According to our previous studies, the imaging doses of DZ-1 and ICG used in this study were 0.5 μmol/kg (10 nmol/mouse) and 10 μmol/kg (200 nmol/mouse) [12,38], respectively. Although there was a 20 times difference in the dose, the fluorescence intensity of DZ-1 per unit area was stronger by one order of magnitude compared to that of ICG, with excellent tumor targeting; even 48 h after administration, NIRF radiant efficiency of the tumor site reached 10^10^ (Figure 3A). However, when ICG was injected into nude mice bearing HCC tumor with the same dose as of DZ-1 (0.5 μmol/kg), NIRF intensity of the tumor site was similar to the background intensity, approximately 10^9^ (Figure 3B). We rarely observed apparent fluorescence, suggesting a strong dose-response relationship. These results were present in both the HCC mouse xenograft model and the rabbit liver tumor model.

Gemcitabine is a deoxycytidine analog that has shown a wide range of antitumor activity [39,40], but its limited benefits are due to the ineffective delivery of therapeutic agents to the tumor tissues as well as undesired side effects elicited by nonspecific biodistribution [41,42]. Recently, theranostic prodrugs, equipped with fluorophores as optical reporters, have become useful to monitor the drug delivery and release process [14], by allowing both real-time monitoring of therapeutic effect and reductions in drug dosages, avoiding potential side effects. NIRF dyes, as drug carriers, covalently conjugate to chemotherapeutic agents, facilitating the ability of drugs to penetrate the blood tumor barrier (BTB). It has been reported that NIRG conjugates can penetrate the BBB/BTB to inhibit brain tumors and prostate tumor brain metastases and prolong the survival of mice [10]. In this study, we also demonstrated the exclusive accumulation of the DZ-1 conjugated with gemcitabine (NIRG) in liver tumors of PDX mice, and restricted the growth of HCC effectively (Figure 6).

In summary, the novel heptamethine cyanine dye DZ-1 and derivative dye-drug conjugates (NIRG) are effective and promising new imaging and targeting agents for the detection and treatment of human liver tumors. DZ-1 demonstrated superiority over ICG for HCC imaging by significantly enhanced tumor-targeting specificity in liver xenograft models. Our studies provided insights for a new generation of NIRF imaging agents that can be used in improved HCC detection and diagnosis as supplements for existing clinical diagnostics, especially for intraoperative guidance of liver cancer surgery.

## 4. Materials and Methods

### 4.1. Animals

Male BALB/c nude mice, 6–7 weeks of age, were purchased from Vital River (Beijing, China), bred in an SPF-level barrier environment at the Laboratory Animal Center within the Fourth Military Medical University (FMMU). Male New Zealand rabbits, weighing between 2 and 2.2 kg, was provided by Xi’an Dile Pu Biotechnology (Xi’an, China). Both mice and rabbits were anesthetized with a combination of intravenous ketamine (10 mg/kg) and xylazine (3 mg/kg), and then maintained under isoflurane during surgery and imaging (RC2 Rodent gas anesthesia machine). All animal experiments were approved (4 February 2016) by the animal welfare ethics committee of FMMU (No. 16013).

### 4.2. Clinical Specimens

All clinical liver HCC specimens, including C64003, C34566, and B66873, were obtained from the Department of Hepatobiliary and Pancreaticosplenic Surgery at Xijing Hospital. The histology subtypes were poorly-differentiated hepatocellular carcinomas. The use of human tissue specimens in research was approved by the institutional review board (IRB) of the FMMU. To establish PDX models, fresh human liver cancer specimens were transplanted subcutaneously into nude mice according to a published protocol [19].

### 4.3. Reagents

Human HCC cell line Hep3B2.1-7 (Hep3B) was purchased from the Shanghai Institute of Life Sciences, Chinese Academy of Sciences. Lentivirus-mediated transfection of Luc-Puro cell kit was purchased from Han Heng Biotechnology (Shanghai, China). MEM/EBSS culture media and fetal bovine sera were purchased from Hyclone/Thermo Scientific (Waltham, MA, USA). Heptamethine cyanine compounds DZ-1 and NIRG were provided by Dr. Leland W.K. Chung (Cedars-Sinai Medical Center, Los Angeles, CA, USA). The chemical structures of DZ-1 and NIRG are shown in Supplemental Figure S1A,B. Rabbit VX-2 liver tumor tissue was preserved by our laboratory. Primary antibodies were used as follows: AFP (1:100; BD Bioscience, San Jose, CA, USA), HIF1a (1:100; Abcam, Cambridge, UK), and OATP (1:50; Santa Cruz, Santa Cruz, CA, USA).

### 4.4. The Emission Spectrum of DZ-1

Emission spectra of 10 M DZ-1 were detected by spectrometer. First, the full spectrum excitation of 200–900 nm was selected. Then, the best excitation wavelength of DZ-1 was selected in the full spectrum excitation spectrum. Finally, the emission spectra of DZ-1 were detected by the optimal excitation wavelength (Figure S1C). The optimal excitation wavelength of DZ-1 was 767 nm, and the optimal emission wavelength was 798 nm.

### 4.5. Bioluminescence and NIRF Imaging for Tumor Xenograft Models

Whole-body or organ-specific optical imaging was performed at multiple time points after injection of DZ-1, using the Caliper Lumina II Small animal optical imaging system (PerkinElmer, Waltham, MA, USA) equipped with NIRF filter sets (excitation/emission, 783/840 nm) [18], and calculate the fluorescence intensity per square centimeter of tumor. Bioluminescence (BLI) imaging of tumor xenografts, either in vivo or ex vivo, was performed after mice received d-luciferin (3 mg/mouse, via intraperitoneal delivery). NIRF optical fiber exploration instruments were provided by the Institute of Automation, Chinese Academy of Sciences (ID.140800000099004).

### 4.6. DZ-1 and ICG Uptake by Human HCC Cells

Hep3B was cultured in MEM/EBSS medium, supplemented with 10% fetal bovine serum and 1% penicillin/streptomycin. After 24 h, 20 μM each of ICG or DZ-1 was added. The co-culture was incubated for 30 min and washed three times with PBS, and then cells were allowed to continue growth in the culture vessel. At 1, 3, 6, 12, and 24 h, the cells were subjected to analysis of NIRF dye uptake by a NIRF microscope (Olympus 1 × 71, Olympus, Melville, NY, USA) equipped with a 75 W Xenon lamp and an ICG filter cube (excitation/emission: 750–800/820–860 nm).

### 4.7. Metabolism of NIRF Dyes in Nude Mouse Subcutaneous Xenograft HCC Model

To establish subcutaneous tumor xenograft models, 2 × 10^6^ Hep3B cells were implanted into nude mice. Two weeks later, the mice bearing tumors were injected intravenously with ICG (10 or 0.5 μmol/mouse) or DZ-1 (0.5 or 200 μmol/mouse), respectively. After 12, 24, and 48 h, we measured NIRF intensity at the tumor site and within major organs (including heart, liver, spleen, lung, and kidney) by small animal optical imaging. We compared NIRF intensity value across tissues and organs.

### 4.8. NIRF Dye Uptake by Orthotopic Liver Xenograft Model in Nude Mice

Hep3B cells were transfected with lentiviral vectors containing the luciferase gene, followed by puromycin (0.1 g/mL) selection to ensure a 100% integration rate of luciferase in stable Hep3B2.1-7 cells, which was confirmed by fluorescence microscopy. Then, 2 × 10^6^ Hep3B-Luc cells were subjected to orthotopic liver transplantation into the nude mice. Two weeks later, we detected luciferase signals in the liver of model mice by the same method as 4.5. At the same time, mice bearing tumors were injected with ICG (10 µmol/kg) or DZ-1 (0.5 μmol/kg). After 24 h, the mice were sacrificed, and the NIRF signal intensity of heart, liver, spleen, lung, kidney, and other organs was measured.

### 4.9. Imaging of NIRF Dye in Rabbit Liver Tumor Model

Male New Zealand rabbits were anesthetized with ketamine/xylazine combination. Fresh rabbit liver cancer VX2 tumor tissue was transplanted subcutaneously in rabbits, and a solid tumor was formed after two weeks. A mini-laparotomy was performed in the subxiphoid area of the anesthetized rabbits, exposing the liver. An incision of 1 cm was made in the liver lobe. Then, 3-mm^3^ pieces of rabbit VX2 tumor from subcutaneous tumor were implanted into the hepatic incision. Pressure was applied over the puncture site using a gelatin sponge for approximately 2 min. Abdominal muscles and skin were sutured. Ten days after tumor implantation, the rabbits were intravenously injected with NIRF dye DZ-1 (0.5 μmol/kg) or ICG (10 μmol/kg). Anesthetized rabbits were celiotomized to expose the liver 12 h after dye administration. Liver tumor margins were detected by an NIRF optical fiber exploration instrument [43]. The removed liver was washed with phosphate-buffered saline three times, and optical imaging was performed as previously described. The signal tissue was fixed with 4% formalin for histopathologic analysis.

### 4.10. Statistical Analysis

All values in figures are presented as mean ± standard error of measurement (SEM) for at least three independent experiments. Comparisons between Kaplan-Meier curves were performed using the log-rank test. All other comparisons were analyzed by unpaired two-tailed Student’s *t*-tests. *p* ≤ 0.05 was considered significant.

## Supplementary Materials

Supplementary materials can be found at https://www.mdpi.com/1422-0067/18/6/1332/s1.

## Abbreviations

NIRF	Near infrared fluorescence
ICG	Indocyanine green
HCC	Hepatocellular carcinoma
PDX	Patient-derived xenograft
F/T	Fluorescence intensity/tumor area
NIRG	Analog-gemcitabine conjugate
FMMU	Fourth Military Medical University
BLI	Bioluminescence
