# Correction: Zhang et al. The Application of Heptamethine Cyanine Dye DZ-1 and Indocyanine Green for Imaging and Targeting in Xenograft Models of Hepatocellular Carcinoma. *Int. J. Mol. Sci.* 2017, *18*, 1332

**DOI:** 10.3390/ijms27020860

**Published:** 2026-01-15

**Authors:** Caiqin Zhang, Yong Zhao, He Zhang, Xue Chen, Ningning Zhao, Dengxu Tan, Hai Zhang, Changhong Shi

**Affiliations:** Laboratory Animal Center, the Fourth Military Medical University, Xi’an 710032, China; zhangcaiqin-beibei@163.com (C.Z.); zhaoyong8@aliyun.com (Y.Z.); alwayszhh@163.com (H.Z.); chenxuefamily6@163.com (X.C.); 18729517815@163.com (N.Z.); dgjdg@foxmail.com (D.T.); hzhang@fmmu.edu.cn (H.Z.)

In the original publication [1], there was a mistake in Figure 5C and Figure 6A,B as published. Specifically, in Figure 5C, the image of H&E staining of the fluorescent tissue section treated with DZ-1 (H&E staining, 20×) was mistakenly the same as the image of H&E staining of the fluorescent tissue section treated with ICG (H&E staining, 20×) from Figure 5A. In Figure 6A, the C34566, OATP panel was incorrectly mixed up during the figure assembly, and the wrong panels were uploaded. Similarly, in Figure 6B, the BSP panels were mistakenly mixed up, and the incorrect panels were uploaded. The corrected Figure 5C (H&E staining, 20×, treated with ICG), Figure 6A (C34566, OATP panel), and Figure 6B (BSP panels) appear below. The authors state that the scientific conclusions are unaffected. This correction was approved by the Academic Editor. The original publication has also been updated. We provide the complete corrected figures below.

## Figures and Tables

**Figure 5:**
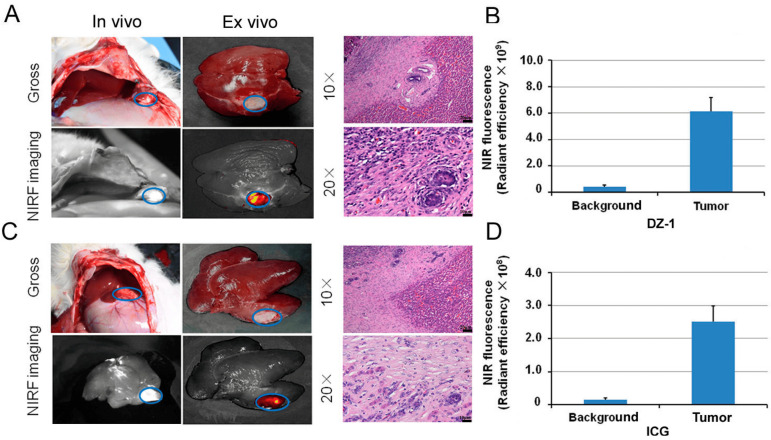
The application of DZ-1 and ICG in surgical exploration. (**A**) DZ-1 imaging using NIRF optical fiber intraoperative guidance in rabbits with VX2 liver cancer (left). Ex vivo NIRF imaging of rabbit liver cancer using small-animal optical imaging system (right). Hematoxylin-eosin (H&E) staining of the fluorescent tissue sections; (**B**) NIRF intensity/tumor area (per cm^2^) of DZ-1 uptake in liver tumor of rabbit; (**C**) ICG imaging using NIRF optical fiber intraoperative guidance in rabbits with VX2 liver cancer (left). Ex vivo NIRF imaging of rabbit liver cancer using small animal optical imaging system (right). H&E staining of the fluorescent tissue sections. 10× and 20×, Scale bars, 20 μm; (**D**) NIRF intensity/tumor area (per cm^2^) of ICG uptake in liver tumor of the rabbits. Blue circle indicate the tumor location.

**Figure 6:**
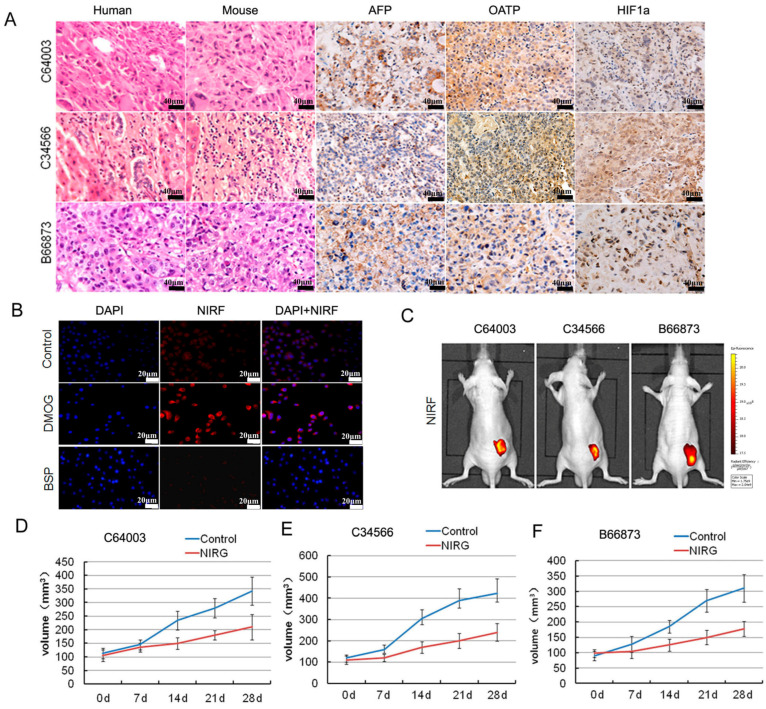
Imaging and targeting of DZ-1 drug conjugate in a liver cancer patient-derived xenograft (PDX) model. (**A**) H&E, NIRF, and immunohistochemistry (IHC) analyses of liver cancer tissues derived from both PDX mouse models and original patient samples. Original magnification: 400×; scale bars represent 20 μm; (**B**) DZ-1 dye uptake by Hep3B cells with a prior exposure to either HIF1α stabilizers (DMOG), or OATP inhibitor (BSP). Scale bar, 50 μm; (**C**) NIRF optical imaging of PDX models established by implanting 3 different human liver cancer specimens to subcutaneous of nude mice. Strong fluorescent signal was detected at subcutaneous tumor site; (**D**–**F**) Inhibition of NIRG on the tumor growth from liver cancer PDX mouse, including C64003, C34566, and B66873. d represents the treatment time.
